# Strategies for Maximising Lung Utilisation in Donors After Brain and Cardiac Death: A Narrative Review

**DOI:** 10.3390/jcm14155380

**Published:** 2025-07-30

**Authors:** Carola Pergolizzi, Chiara Lazzeri, Daniele Marianello, Cesare Biuzzi, Casagli Irene, Antonella Puddu, Elena Bargagli, David Bennett, Chiara Catelli, Luca Luzzi, Francesca Montagnani, Francisco Del Rio Gallegos, Sabino Scolletta, Adriano Peris, Federico Franchi

**Affiliations:** 1 Department of Medical Science, Surgery and Neurosciences, Cardiothoracic and Vascular Anesthesia and Intensive Care Unit, University Hospital of Siena, 53100 Siena, Italy; carola.pergolizzi@gmail.com (C.P.); d.marianello83@gmail.com (D.M.); irecas91@yahoo.it (C.I.); lellapud@hotmail.it (A.P.); 2 Regional Center for Transplant Coordination, 50100 Florence, Italy; lazzeri.ch@gmail.com (C.L.); adriano.peris@gmail.com (A.P.); 3 Department of Medical Science, Surgery and Neurosciences, Urgency-Emergency Anaesthesia and Intensive Care Unit, University Hospital of Siena, 53100 Siena, Italy; cesare1205@gmail.com (C.B.); sabino.scolletta@unisi.it (S.S.); 4 Respiratory Diseases Unit, Department of Medicine, Surgery and Neurosciences, University Hospital of Siena, 53100 Siena, Italy; bargagli2@unisi.it (E.B.); david.btt@gmail.com (D.B.); 5 Thoracic Surgery and Lung Transplant Unit, University Hospital of Siena, 53100 Siena, Italy; chiara.catelli2@unisi.it (C.C.); luca.luzzi@unisi.it (L.L.); 6 Department of Medical Sciences, Infectious and Tropical Diseases Unit, University Hospital of Siena, 53100 Siena, Italy; francesca.montagnani@unisi.it; 7 Department of Medical Biotechnologies, University of Siena, 53100 Siena, Italy; 8 Oficina Regional de Coordinación de Trasplantes, Servicio Madrileño de Salud, Consejería de Sanidad de la Comunidad de Madrid, 28009 Madrid, Spain; franciscojose.rio@salud.madrid.org

**Keywords:** lung transplantation, donor lung management, donors after brain death (DBD), donors after circulatory death (DCD), ex vivo lung perfusion (EVLP), protective ventilation

## Abstract

Lung transplantation remains the standard of care for end-stage lung disease, yet a persistent gap exists between donor lung availability and growing clinical demand. Expanding the donor pool and optimising donor lung management are therefore critical priorities. However, no universally accepted management protocols are currently in place. This narrative review examines evidence-based strategies to improve lung utilisation across three donor categories: donors after brain death (DBD), controlled donors after circulatory death (cDCD), and uncontrolled donors after circulatory death (uDCD). A systematic literature search was conducted to identify interventions targeting lung preservation and function, including protective ventilation, recruitment manoeuvres, fluid and hormonal management, and ex vivo lung perfusion (EVLP). Distinct pathophysiological mechanisms—sympathetic storm and systemic inflammation in DBD, ischaemia–reperfusion injury in cDCD, and prolonged warm ischaemia in uDCD—necessitate tailored approaches to lung preservation. In DBD donors, early application of protective ventilation, bronchoscopy, and infection surveillance is essential. cDCD donors benefit from optimised pre- and post-withdrawal management to mitigate lung injury. uDCD donor lungs, uniquely vulnerable to ischaemia, require meticulous post-mortem evaluation and preservation using EVLP. Implementing structured, evidence-based lung management strategies can significantly enhance donor lung utilisation and expand the transplantable organ pool. The integration of such practices into clinical protocols is vital to addressing the global shortage of suitable lungs for transplantation.

## 1. Introduction

Lung transplantation has become the standard therapy for end-stage lung disease; however, a persistent gap remains between the number of lung transplants each year and the growing number of patients on the waiting list. An increased number of patients suffering from chronic obstructive pulmonary disease (COPD) and interstitial lung disease need lung transplantation, and COPD is still considered as the third cause of death worldwide according to the World Health Organization. Other indications to lung transplant include cystic fibrosis, pulmonary hypertension, and some rheumatic diseases directly affecting the lung. Expanding the organ donor pool is therefore a major priority in lung transplantation to improve survival and decrease mortality of this growing group of patients facing terminal lung disease. Despite efforts to increase donor availability, the utilisation rate of potential lung donors remains relatively low. Optimising donor lung management could improve lung utilisation rates in both donors after brain death (DBD) and donors after circulatory death (DCD), including both uncontrolled (uDCD) and controlled (cDCD) donation [1,2]. At present, DBD remains the primary donor lung source for transplantation worldwide [3]. Currently, there is no widely accepted standardised protocol for the management of potential lung donors. European governmental policies for lung procurement are primarily guided by Directive 2010/53/EU, which defines quality and safety standards for organs eligible for transplantation. Donor selection, organ traceability, and reporting of adverse events represent the guideline items. However, organ procurement faces several substantial obstacles, not only represented by organ shortage but also public perception, social factors and ethical concerns.

This narrative review aims to summarise current knowledge on lung management in donors across all categories (DBD, uDCD, and cDCD) with the goal of improving lung utilisation rates and addressing the ongoing shortage of transplantable lungs.

## 2. Materials and Methods

A systematic literature search was conducted in PubMed, Embase, and the Cochrane Library using the keywords “lung transplantation and uncontrolled donation after circulatory death”, “lung transplantation and controlled donation after circulatory death”, and “lung transplantation and donors after brain death”. According to the narrative intent of the review, we did not follow formal inclusion/exclusion criteria or a predefined selection process. However, we prioritised studies that were relevant to clinical practice and donor management strategies, focusing—whenever possible—on the most recent literature, with the aim to offer a centred and up-to-date overview of key evidence in the field. The search was limited to studies published in English and excluded case reports, animal studies, and studies lacking original data. Studies were included if they investigated donor management strategies aimed at improving lung utilisation, such as protective ventilation strategies, lung recruitment manoeuvres, fluid management, hormonal resuscitation, and ex vivo lung perfusion (EVLP). The primary outcomes of interest were lung function, lung suitability for transplantation, and post-transplant complication rates. Relevant data from the eligible studies were synthesised narratively to highlight best practices for lung preservation in DBD, uDCD, and cDCD donors.

## 3. Results

### 3.1. Effects of Brain Death on Lung Physiology

#### 3.1.1. Blast Injury Theory

The impairment of lung function following brain death (BD) is primarily related to haemodynamic alterations that affect heart–lung interactions. The massive sympathetic nervous system activation triggered by BD increases left atrial pressure and pulmonary capillary pressure, resulting in pulmonary vasoconstriction and endothelial damage [4]. Consequently, pulmonary capillary permeability increases, contributing to lung dysfunction [5]. These pathophysiological changes are explained by the so-called “blast injury theory,” which proposes that a combination of hydrostatic and high-permeability mechanisms leads to the development of neurogenic pulmonary oedema [6]. Additionally, the intense coronary vasoconstriction induced by the catecholamine storm, without a proportional increase in myocardial oxygen delivery, may result in sub-endocardial ischaemia, further impairing pulmonary function [4,5].

#### 3.1.2. Inflammatory Cascade and Its Consequences: “Double-Hit Model” and “Triple-Hit Hypothesis”

Recent evidence challenges the “blast injury theory,” suggesting that systemic inflammation plays an additional and amplifying role in post-brain death pulmonary dysfunction. Following brain death, intracranial production of pro-inflammatory cytokines (IL-1, IL-6, TNF-α, ICAM-1) increases, releasing inflammatory mediators into the systemic circulation [6]. Notably, DBD donors display significantly higher systemic inflammation compared to DCD donors, as evidenced by elevated cytokine plasma levels in pre-transplant samples. Specifically, IL-6 levels are higher in DBD donors, along with increased IL-10 and IL-8 [3,7]. Moreover, elevated TNF-α plasma levels in donors have been associated with a greater incidence of primary graft dysfunction post-transplantation [7].

This intricate biochemical cascade following brainstem death has led to the formulation of the “double-hit model.” The first hit is driven by the catecholamine surge and the systemic inflammatory response. This inflammatory environment renders the lung more vulnerable to subsequent injurious stimuli, constituting the second hit. Among these second hit factors, mechanical ventilation plays a pivotal role. Particularly, the use of high tidal volumes to maintain normocapnia combined with inadequate positive end-expiratory pressure (PEEP) to correct hypoxemia can exacerbate lung injury. Additional contributing factors include infections, transfusions, and surgical interventions, all of which may worsen lung function in DBD donors. The interplay of these insults can create a vicious cycle of lung injury [6]. Furthermore, the abnormal activation of the coagulation system due to the systemic inflammatory response leads to a prothrombotic state characterised by increased fibrin formation, reduced fibrinolysis, and heightened platelet activation. This prothrombotic condition promotes the formation of microthrombi within the donor lungs, potentially leading to further deterioration in lung function [8].

Recently, a “triple-hit hypothesis” has been proposed, with the third hit represented by alterations in the gut–lung axis. In DBD donors, damage to the intestinal mucosa and increased intestinal permeability have been observed, leading to gut microbiota dysbiosis [9]. Changes in gut microbiome composition appear to be linked to altered immune responses and airway homeostasis, potentially contributing to new or worsening lung injury [10].

#### 3.1.3. Ventilator-Induced Lung Injury (VILI)

The role of mechanical ventilation in worsening lung function in DBD donors can be summarised as follows:

(a) The presence of pulmonary oedema and a pro-inflammatory environment in the DBD lung reduces its tolerance to the mechanical stress imposed by injurious ventilatory strategies. In particular, the use of high tidal volumes and high respiratory rates, combined with a low PaO_2_/FiO_2_ ratio, has been identified as an independent predictor of early acute lung injury (ALI) within the first 72 h of mechanical ventilation. These factors, along with major underlying risk factors, such as aspiration, pneumonia, and lung contusion, as well as certain treatment variables, increase susceptibility to lung injury in patients with severe brain injury [11].

(b) The three primary mechanisms contributing to the development of VILI include volutrauma and barotrauma from excessive lung overdistension during mechanical ventilation, increasing alveolar–capillary permeability. Another mechanism is atelectrauma, which occurs due to the repeated recruitment and derecruitment of collapsed alveoli, further exacerbating lung injury. Additionally, biotrauma plays a crucial role, as mechanical ventilation induces an inflammatory response, amplifying pulmonary dysfunction and further damaging tissue [6].

#### 3.1.4. Apnoea Testing

Apnoea testing may represent an additional harmful stimulus for lung injury, particularly when performed using passive air insufflation inside the endotracheal tube, which can lead to auto-PEEP. The presence of auto-PEEP and air trapping is well known to increase the risk of barotrauma and further compromise lung function [12]. In patients with severe traumatic brain injury, the incidence of ALI is notably high, estimated at 20–30%. Its occurrence follows a bimodal distribution, with an early peak around days 2–3 after the initiation of mechanical ventilation and a late peak around days 7–8, the latter is often associated with the development of concurrent pneumonia [6].

#### 3.1.5. Aspiration Pneumonia

Aspiration pneumonia is a common concern in potential lung donors and is typically assessed through bronchoscopy or CT imaging, particularly by identifying infiltrates in the right lower lobe. Recent studies have proposed the measurement of bile acids in airway bronchial wash samples as a potential marker of aspiration, with higher levels correlating with lower transplant suitability, reduced performance during EVLP, and worse post-transplant outcomes [13]. Due to concerns that aspiration may negatively impact recipient allograft function, the use of lungs from donors affected by aspiration is generally discouraged. However, conflicting evidence exists. Nunley et al. reported that the presence of bile salts in donor lungs does not significantly compromise allograft function and does not increase mortality within the first year post-transplantation [14]. Given these discrepancies, further research is needed to establish clear recommendations regarding the transplant suitability of lungs affected by aspiration.

Main pathophysiological mechanisms underlying brain death are summarised in Table 1.

### 3.2. Management of the DBD Lung

#### 3.2.1. Protective Mechanical Ventilation

A lung management strategy in DBD donors can be established based on available evidence and pathophysiological mechanisms.

The adoption of a lung-protective ventilatory strategy is now considered essential, as it helps prevent alveolar overstretch and end-expiratory collapse while promoting alveolar recruitment and maintaining lung integrity. A randomised controlled trial by Mascia et al. showed that a protective ventilatory approach, characterised by tidal volumes of 6–8 mL/kg of predicted body weight, PEEP of 8–10 cm H_2_O, apnoea tests performed using positive airway pressure, a closed circuit for airway suction, and recruitment manoeuvres performed by doubling ventilation with low tidal volumes for 10 breaths after any disconnection, has been shown to increase the number of lung donors meeting eligibility criteria for transplantation compared to a conventional ventilatory strategy (which involves tidal volumes of 10–12 mL/kg PBW, PEEP of 3–5 cm H_2_O, apnoea tests performed by ventilator disconnection, and a closed circuit for airway suction) [15]. The optimal PEEP level in lung donors remains uncertain. The Spanish consensus document on lung donor management recommends a minimum PEEP of 5 cm H_2_O, though a value of ≥8 cm H_2_O is suggested as ideal [16].

In their review, Meyfroidt et al. proposed a protective ventilatory strategy consisting of a tidal volume of 6–8 mL/kg PBW, PEEP of 8–10 cm H_2_O, a closed circuit for tracheal suction, alveolar recruitment manoeuvres after any disconnection, and the use of continuous positive airway pressure (CPAP) during apnoea testing. This approach has been shown to increase the number of eligible and transplanted lungs without negatively affecting the procurement of other organs [4].

To prevent oxygen toxicity, the minimum FiO_2_ necessary to maintain a PaO_2_ above 100 mmHg should be used. Additionally, respiratory targets should include a physiological pH (7.35–7.45), oxygen saturation (SpO_2_) above 95%, and partial pressure of carbon dioxide (PaCO_2_) between 35–40 mmHg [4].

#### 3.2.2. Recruitment Manoeuvres

According to an influential review, recruitment manoeuvres should be routinely performed in donors when PaO_2_/FiO_2_ remains below 300 mmHg, or in the presence of atelectasis or pulmonary oedema of any origin [17].

Currently, one of the most widely adopted ventilatory protocols for DBD donors is the one proposed in their prospective study by Minambres et al. in 2014 [18], which includes apnoea testing with the ventilator in CPAP mode, mechanical ventilation with PEEP set between 8 and 10 cm H_2_O, and tidal volumes of 6–8 mL/kg. Additionally, this protocol recommends performing recruitment manoeuvres once per hour and after any disconnection from the ventilator, as well as alveolar recruitment through controlled ventilation with decremental PEEP.

#### 3.2.3. Fluid Strategy in Potential Lung Donors

Donor management guidelines emphasise the importance of preventing or immediately correcting hypovolaemia to maintain adequate organ perfusion and ensure the viability of transplantable organs. Euvolemia is the primary therapeutic goal, achieved through volume replacement therapy, in which isotonic crystalloid solutions are the preferred choice [4,19]. Among these, balanced salt solutions, such as Ringer’s lactate, may be superior to normal saline, as they do not induce hyperchloremic acidosis [19].

Conversely, starch-based synthetic colloids should be avoided due to their adverse effects in critically ill patients especially on renal epithelial cells, which may contribute to early graft dysfunction in transplanted kidneys [4,19].

It has long been recognised that when lungs are considered for transplantation, fluid administration should be carefully controlled [20]. Maintaining a neutral or slightly negative fluid balance, achieved through judicious administration of crystalloids and diuretics, is associated with improved oxygenation and better lung donor outcomes [1]. Tight regulation of fluid status is essential to prevent pulmonary oedema while ensuring adequate systemic perfusion [2]. In this regard, restrictive fluid management strategies have been shown to enhance lung graft harvesting [19].

Similarly, donor blood transfusion has been identified as a potential risk factor for primary graft dysfunction, likely due to mechanisms similar to those of transfusion-related acute lung injury [2]. However, there are no current universal recommendations on transfusion targets in donor management [4].

#### 3.2.4. Bronchoscopy

Fiberoptic bronchoscopy is an essential tool in assessing lung suitability in potential donors [2,18]. Bronchoscopic evaluation allows confirmation of airway integrity and anatomy, which is crucial for successful surgical anastomosis of the major bronchi. Additionally, it facilitates the exclusion of aspiration of gastric contents, neoplasia, or foreign materials in the airways and enables the screening of potentially harmful microorganisms in respiratory secretions. However, airway erythema or secretions alone do not preclude organ retrieval, and influential reviews suggest that bronchoalveolar lavage should be routinely performed to guide antimicrobial treatment for lower respiratory tract infections and improve oxygenation by removing airway obstructions [1,2,18,21].

Bronchoscopy at ICU admission is recommended for potential lung donors, as well as for critically ill patients when aspiration pneumonia is suspected. Patients with acute brain injury requiring mechanical ventilation are at high risk of ventilator-associated pneumonia (VAP), and this protocol could potentially be extended to DBD donors, although further studies are needed [22].

According to current literature, lungs from donors colonised with multidrug-resistant (MDR) bacteria can be safely transplanted, provided that recipients receive prompt, preventive, and tailored antibiotic therapy, including both systemic and nebulised antibiotics [23,24]. However, caution is advised in cases of MDR Klebsiella pneumoniae, as active infection with MDR organisms, invasive fungal infections, or mycobacterial infections remains a relative contraindication to lung transplantation. In contrast, active Mycobacterium tuberculosis infection is considered an absolute contraindication [2,24]. Donor bacteraemia or sepsis should not be regarded as an absolute contraindication to organ donation unless strongly supported by microbiological and imaging evidence of uncontrolled infection. In such cases, lungs may still be considered for transplantation if appropriate antibiotic therapy has been administered for at least 48 h prior to procurement [2,25].

#### 3.2.5. Endotracheal Tube Management and Imaging

To prevent lung derecruitment, clamping the endotracheal tube (ETT) is a common practice in many transplant centres. ETT clamping at end-inspiration, with tidal volume reduced by half, can help minimise airway pressure loss under PEEP and reduce the risk of high alveolar pressures upon ventilator reconnection [26]. Among available methods, the Extra Corporeal Membrane Oxygenation clamp has been identified as the most effective in maintaining alveolar recruitment and improving oxygenation. For this reason, endotracheal tube clamping is recommended in potential DBD lung donors [27].

In terms of lung imaging, a clear chest X-ray is considered a prerequisite for donor lung acceptability. However, an abnormal chest X-ray alone should not exclude a lung from recovery, as it may still be suitable for transplantation. Chest X-rays are useful in estimating total lung capacity and detecting vascular congestion, oedema, infiltrates, and contusions, which may indicate the need for further imaging, such as a CT scan. Current practices in the USA and EU involve performing at least one CT scan to assess donor lungs and identify abnormalities not visible on a chest X-ray [2].

#### 3.2.6. PaO_2_/FiO_2_ Ratio and the “40-100 Test” in Donor Lung Assessment

The PaO_2_/FiO_2_ ratio is a well-validated criterion in donor scoring systems and plays a crucial role in assessing lung suitability for transplantation. To evaluate lung function, each DBD donor should undergo the “40–100 test,” which involves comparing PaO_2_/FiO_2_ ratios obtained from two different arterial blood gas analyses. The first sample is taken at 40% FiO_2_ (representing the basal oxygenation status), while the second is collected at 100% FiO_2_ (reflecting maximal oxygenation).

The rationale behind this test is to detect and quantify the presence of an intrapulmonary shunt, which represents a significant functional impairment in ALI. Shunting occurs when pulmonary blood flow bypasses non-ventilated alveoli, leading to impaired oxygenation despite increased inspired oxygen concentration. The 40–100 test helps to unmask this condition, providing critical information for lung donor evaluation and guiding the decision-making process regarding lung suitability for transplantation.

#### 3.2.7. Pronation

Atelectasis is one of the primary reversible causes of hypoxemia following brain death. In a prospective trial [28], ventilation in the prone position has been shown to effectively reverse atelectasis and rapidly improve oxygenation in hypoxic DBD donors.

In DBD donors, atelectasis often develops due to the absence of spontaneous respiration, cough reflex, and respiratory drive, leading to ventilation–perfusion mismatch and hypoxemia. Additional donor-related risk factors, including mechanical ventilation, prolonged supine positioning, and obesity, further contribute to atelectasis [29]. To counteract this, aggressive lung management strategies incorporating recruitment manoeuvres, bronchoscopy, chest percussion, and lung-protective ventilation can be employed to improve donor lung suitability for transplantation.

However, in cases in which severe haemodynamic instability, abdominal injuries, or multiple trauma are absent, prone positioning may not be an option to be carefully considered [29]. As an alternative, lateral rotation therapy—which involves positioning the donor intermittently or continuously from side to side using a programmable bed—may be considered.

According to a retrospective observational study, the implementation of a rotational positioning protocol in DBD donors has been shown to significantly increase the PaO_2_/FiO_2_ ratio and improve the rate of successful lung donations, making it a safer and more feasible alternative when the donor is haemodynamically unstable [30].

### 3.3. Lung Transplantation from Donors After Controlled Cardiac Death (cDCD)

According to the Maastricht classification, class III cDCD refers to patients for whom the withdrawal of life support is a planned decision based on diagnostic and prognostic considerations. These donors are commonly referred to as controlled DCD donors [31].

Current evidence suggests that lung transplant recipients from cDCD donors experience similar outcomes to those receiving lungs from DBD donors, both in terms of survival rates and graft function [32,33].

#### 3.3.1. Lung Injury in cDCD—Potential Mechanisms

##### Warm Ischaemia

Warm ischaemia is defined as the time interval from the cessation of circulation until cold perfusion is initiated. During this period, pulmonary blood vessels remain filled with warm, non-circulating blood, which can form a thrombus within the lung vasculature. This process contributes to increased vascular resistance, reduced vascular compliance, and impaired gas exchange, potentially affecting lung viability for transplantation [32,34].

##### Prolonged Mechanical Ventilation

In cDCD donors, the overall period of mechanical ventilation is typically longer compared to DBD donors, regardless of the underlying cause of death [35]. While prolonged ventilation alone is not an exclusion criterion for lung donation [21], cDCD donors are considered at higher risk for complications related to extended mechanical ventilation, including VILI and VAP.

Prolonged hospitalisation also represents an additional risk factor due to prolonged intensive care procedures, increasing susceptibility to infections [14]. However, an important distinction exists: unlike DBD donors, cDCD donors do not experience the inflammatory cascade triggered by brain death. The absence of systemic inflammatory mediators may actually be advantageous for lung transplantation, potentially reducing the severity of ischaemia–reperfusion injury and improving post-transplant graft function [36].

### 3.4. Management of Lungs from cDCD

#### 3.4.1. Protective Mechanical Ventilation

A key difference between death following cardiac arrest after life support withdrawal (cDCD) and death due to neurological criteria (DBD) is the longer period typically spent before the decision to withdraw life support in cDCD donors. This extended period offers an opportunity to optimise lung recovery from concomitant injuries and enhance the effects of protective ventilation strategies, potentially improving organ suitability for transplantation. However, limited literature exists on the specific management of cDCD donor lungs, though the principles of protective mechanical ventilation remain similar to those applied in DBD donors.

Beyond the pre-mortem phase, mechanical ventilation remains crucial even post-mortem, as ventilated lungs can better tolerate prolonged circulatory arrest while maintaining cellular viability. This situation is made possible by passive oxygen diffusion through the alveolar wall, which continues to provide oxygen to pulmonary epithelial cells even after circulatory arrest [36].

To minimise infectious risks, general recommendations for donor management include performing blood cultures and respiratory secretion Gram stains at organ retrieval. In cases of active bacterial infections, antimicrobial therapy should be administered for at least 48 h before procurement to optimise lung suitability [37].

#### 3.4.2. Recruitment Manoeuvres

cDCD donors can benefit from applying recruitment manoeuvres and tend to tolerate them well, similarly to DBD donors. A recent experimental study by Niman et al. demonstrated that recruitment manoeuvres in the absence of circulation may protect against atelectasis. These findings suggest that inflation procedures in cDCD donors can be safely executed even after cardiac arrest, potentially optimising lung recruitment and preservation [38].

#### 3.4.3. Pronation

In cases in which oxygenation parameters remain unsatisfactory, DCD donors might theoretically benefit from prone positioning, although no clinical data currently support this approach. As an alternative, lateral positioning could serve as a viable option for improving lung aeration and reducing atelectasis.

Experimental evidence from animal models suggests that prone positioning during warm ischaemia can significantly enhance lung quality and graft preservation, even under conditions of absent perfusion and ventilation. These findings indicate a potential application of this technique in human DCD donors, though further studies are needed to confirm its clinical feasibility [39].

#### 3.4.4. Bronchoscopy

The rationale for bronchoscopy in cDCD donors is similar to that in DBD donors, primarily to exclude major anatomical abnormalities, detect macroscopic aspiration, and identify potentially harmful pathogens in respiratory secretions. Bronchoscopy should be conducted as early as possible to allow sufficient time for diagnostic results to become available and to adjust antimicrobial therapy if necessary. However, at a minimum, bronchoscopy should be performed at the time the donor enters the operating room to ensure adequate airway assessment and preparation for lung procurement, as indicated by Egan et al. in their review [33].

#### 3.4.5. Endotracheal Tube Management

The practice of clamping the ETT to prevent lung derecruitment and monitoring cuff pressure to minimise aspiration is advisable in DCD donors. This strategy should be implemented not only after cardiac arrest but also throughout the entire ICU care period to optimise lung preservation. Following the declaration of death, the ETT should remain in place and be inspected to ensure it remains inflated. If necessary, re-insertion may be performed to facilitate lung ventilation and reduce aspiration risk. Once the donor reaches the operating room, they should be reconnected to the ventilator, and mechanical ventilation should be resumed to optimise lung function before procurement [33].

### 3.5. Lung Transplantation from Donors After Uncontrolled Cardiac Death (uDCD)

uDCD represents a valuable source of donor lungs, and since Steen’s 2001 landmark case in Sweden, lungs from uDCD donors have been successfully transplanted in several European countries, including France, the Netherlands, Spain, and Italy, with promising outcomes [40]. The lung’s unique physiology allows it to tolerate periods of circulatory arrest, as it does not rely solely on blood perfusion for aerobic metabolism but rather on passive oxygen diffusion through the alveoli [41]. Unlike other organs, the lung has relatively low metabolic demands and retains a local oxygen reserve within the alveoli. Egan et al. [42] experimentally demonstrated the feasibility of transplanting donor lungs after cardiac arrest with an absence of circulation for up to two hours. In contrast to brain-dead and controlled DCD donors, lung function in uDCD donors cannot be assessed before death, though viability can be effectively evaluated post-mortem using EVLP. In their case report in 2016, Valenza et al. [43] reported that the lungs were preserved in uDCD donors through recruitment manoeuvres, CPAP, and protective mechanical ventilation, followed by EVLP. In contrast, the Spanish experience favoured lung maintenance via topical cooling. The rationale behind these strategies is supported by experimental models, which have identified the prevention of alveolar collapse as a critical factor in mitigating reperfusion injury, independent of continuous oxygen supply [43,44]. While in situ lung cooling may provide superior protection, its application in uDCD settings is logistically challenging and time-consuming. Conversely, the “protective ventilation technique” is simpler, more widely applicable, and could facilitate the broader use of uDCD lung donors [40]. To date, clinical evidence regarding lung transplantation following protective ventilation as a preservation strategy remains limited but encouraging. At present, experiences with “uDCD only lung programs” are circumscribed, represented by case series mostly from Spanish centres. Despite the limited number of patients enrolled [40], all investigations documented a good long-term graft survival rate. Valvidia et al. [45] compared the outcomes of 38 recipients of lungs from uDCD donors with those of 292 recipients of lungs from DBD donors, and no differences were observed between the two groups in both early and long-term outcomes (ICU and hospital stay, primary graft dysfunction and chronic graft dysfunction). On the contrary, the two groups differed in global survival at 1, 5, and 10 years (71.1%, 50.8%, and 16.5% versus 75%, 58.4%, and 38.1%, respectively). It is worth noting that EVLP was performed only in the 21%. Similar results were also reported in Canada [46] and Netherlands [47].

According to available evidence and our experience [48], we identified several inclusion and exclusion criteria that are listed in Table 2.

All reported protocols include a witnessed cardiac arrest as an inclusion criterion, with notable differences in preservation times (240 vs. 180 min) and donor age thresholds (<55 years in Spanish protocols and <65 years in Toronto protocols) [46,49,50,51]. Despite these protocol variations, uDCD lungs have yielded promising transplant outcomes, and further optimisation of EVLP techniques may enhance their utilisation, potentially expanding the available donor pool.

Concerning organisational issues, a pivotal key factor for the implementation of an “only lung uDCD” program is the distance between the procurement centre and the lung transplant centre. Whenever this distance is more that 50 kms, the possibility of initiating EVLP at the procurement centre should be considered.

## 4. Discussion

There is a clinical need to improve the utilisation rate from donors. DBD, cDCD, and uDCD donors present specific pathophysiological characteristics, as resumed in Table 3. In the absence of a standardised and widely accepted protocol on lung maintenance and considering available evidence and our own experience, we have elaborated a strategy according to the different types of donors, as depicted in Table 4.

The optimisation of lung donor management is crucial to maximising graft viability and expanding the available donor pool. Each type of donation—DBD, cDCD, and uDCD—presents distinct physiological challenges that require tailored preservation strategies. While DBD donors allow for controlled interventions from ICU admission, cDCD donors require a delicate balance between ventilatory support and ischaemia prevention before the withdrawal of life support. uDCD donors, on the other hand, undergo a period of uncontrolled ischaemia, necessitating post-mortem assessment and rescue strategies such as EVLP. In all cases, a structured approach including bronchoscopy, positioning strategies, and infection surveillance plays a fundamental role in optimising lung function and ensuring transplant success.

While protective ventilation and recruitment manoeuvres should be performed in all types of donors, there are differences among the various donor types.

In potential DBD lung donors, bronchoscopy should be performed upon ICU admission to assess airway integrity and clear secretions. Protective ventilation should be initiated early to prevent alveolar damage and atelectasis, with adherence to standardised protocols where feasible. Given the high risk of VAP, routine microbiological surveillance should be implemented to enable early infection detection and guide targeted antimicrobial therapy. Prone positioning may aid in lung recruitment, though its feasibility should be carefully evaluated considering haemodynamic stability. If contraindicated, lateral rotation therapy may serve as an alternative. All interventions should be integrated into an organised donor management strategy to optimise lung function before procurement.

In potential cDCD donors, serial bronchoscopy should be carefully assessed and lateral positioning considered to facilitate lung recruitment and secretion clearance. Before death certification, optimising lung function remains a priority, as prolonged ventilatory support increases the risk of atelectasis and ischaemia. Prone positioning has demonstrated benefits in experimental models, improving oxygenation and mitigating ischaemic injury, suggesting its potential role in clinical practice [39]. Bronchoscopy plays a fundamental role in airway evaluation, aspiration prevention, and microbiological surveillance and should be performed early, ideally before procurement [33]. Infection control strategies require routine blood cultures and respiratory secretion analysis, with antimicrobial therapy reserved for confirmed infections [37]. A structured donor management approach integrating positioning strategies, bronchoscopy, and infection surveillance is critical for ensuring optimal lung viability while minimising unnecessary interventions.

The management of uDCD donor lungs is complex due to uncontrolled warm ischaemia and the need for post-mortem viability assessment. Before death certification, minimising alveolar collapse may improve outcomes, while bronchoscopy at retrieval helps assess airway integrity [41,43]. Microbiological surveillance is crucial, with antimicrobial therapy guided by post-procurement findings [43]. After death certification, preservation strategies vary, with protective ventilation and topical cooling both employed. EVLP enables functional graft assessment and optimisation. Proper endotracheal tube management and lung recruitment remain essential to maximising uDCD lung viability [43].

Although substantial progress has been made, current evidence on lung donor optimisation strategies is still limited by heterogeneity in clinical protocols, small sample sizes, and variability in outcome measures. Further high-quality, multicentre, and prospective studies are urgently needed to establish standardised best practices. International consensus and updated clinical guidelines will be essential for harmonising donor management strategies and ultimately enhancing recipient outcomes in lung transplantation.

## 5. Conclusions

Potential lung DBD donors benefit from the ability to implement lung-protective strategies early, minimising ischaemia and optimising oxygenation before procurement. cDCD donors introduce challenges due to the need for lung protection during the withdrawal phase, requiring a careful balance between recruitment and preventing prolonged ventilatory exposure. In contrast, uDCD donors face the greatest ischaemic burden, making rapid retrieval and post-mortem assessment critical. Despite these differences, incorporating all three types of lung donation into clinical practice significantly expands the donor pool. The growing success of EVLP has further increased the feasibility of utilising lungs from cDCD and uDCD donors, reinforcing the importance of a comprehensive donor management strategy that integrates all available sources to meet the increasing demand for lung transplantation.

Intensive care strategies for lung maintenance are essential to improving lung suitability for transplantation and must account for the distinct pathophysiological mechanisms of lung injury in each type of donor. In DBD and cDCD donors, lung injury prevention is paramount, with infection surveillance and protective ventilation serving as fundamental components that should be initiated upon ICU admission. Bronchoscopy should be a mandatory practice in all patients with acute cerebral lesions, particularly those at high risk for brain death, and should be prioritised when aspiration pneumonia is suspected. In uDCD, where warm ischaemia is unavoidable, rapid lung retrieval and post-mortem assessment, including EVLP, are critical to maximising graft viability. A comprehensive and tailored approach to each donor type is essential to expanding the lung donor pool and improving transplant outcomes.

## Figures and Tables

**Table 1:** Pathophysiological mechanisms in donors after brain death.

Mechanism	Description
Blast Injury Theory	Combination of hydrostatic and high-permeability mechanisms leading to neurogenic pulmonary oedema
Double-Hit Model	Initial injury from catecholamine surge and systemic inflammation, followed by secondary insults like ventilation and infections
Triple-Hit Hypothesis	Involvement of gut-lung axis through intestinal permeability changes, microbiome alterations, and immune modulation
Ventilator-Induced Lung Injury (VILI)	Volutrauma, barotrauma, and atelectrauma contributing to lung injury via overdistension and repetitive collapse
Apnoea Testing	Risk of auto-PEEP development, leading to barotrauma and increased airway pressure
Aspiration Pneumonia	Inflammation from aspiration linked to reduced transplant suitability, with conflicting evidence on its impact on outcomes

**Table 2:** Inclusion and exclusion criteria and relative contraindications for uDCD 2 donors enrolment. We highlight that the presence of aortic acute dissection does not represent a contraindication for the assessment of a potential uDCD lung donor.

*Inclusion Criteria*	*Exclusion Criteria*	*Relative Contraindication*
Age 18–65 years	Unidentified donor	BMI > 40 kg/m^2^
Witnessed cardiac arrest	Failure to manage airways	Smoking
Identified donor	Ab ingestis pneumonia	Chronic heart disease
Managed airways (intubation or laryngeal mask)	Chest trauma	
	Airway bleeding	

**Table 3:** Main pathophysiological, aspects of donors after brain death and donors after cardiac death (controlled and uncontrolled).

	DBD	cDCD	uDCD
**Pathophysiology**	Inflammatory and catecholaminergic response with maintained ventilation and oxygenation	1. Prolonged mechanical ventilation: VILI and infection 2. Warm ischemia	Unexpected cardiac arrest, prolonged warm ischemia, and increased hypoxia during transport to ICU or operating room. No previous lungprotective strategies

**Table 4:** Practical summary of main recommendations.

Donor	Ventilation			Recruitment Manoeuvres	Timing for Prone Position	Timing for Bronchoscopy
	TV	PEEP	FiO_2_			
**DBD**	8 mL/kg	8 cm H_2_O	the lowest to keep SpO_2_ > 95%	to be performed regularly to improve oxygenation	routinely when PaO_2_/FiO_2_ < 300 mmHg or in case of atelectasis or pulmonary oedema; lateral semi-recumbent position as an alternative	routinely to assess airway integrity and guide antimicrobial treatment
**cDCD**	6–8 mL/kg	8–10 cm H_2_O	the lowest to keep SpO_2_ > 95%		prior to cardiac arrest, according to donor status	pre-retrieval or post-withdrawal, depending on clinical status
**uDCD**	6–8 mL/kg	10 cm H_2_O	50%		to be considered during EVLP	at the beginning of retrieval

## Abbreviations

The following abbreviations are used in this manuscript:

DBD	Donor after brain death
DCD	Donor after circulatory death
EVLP	Ex vivo lung perfusion
VILI	Ventilator induced lung injury
PEEP	Positive end expiratory pressure
TV	Tidal volume
ICU	Intensive care unit
CPAP	Continuous positive airway pressure
VAP	Ventilator-associated pneumonia
MDR	Multidrug resistant
